# Comparing high‐dose cisplatin with cisplatin‐based combination chemotherapy in definitive concurrent chemoradiation setting for locally advanced head and neck squamous cell carcinoma (LAHNSCC)

**DOI:** 10.1002/cam4.2139

**Published:** 2019-04-09

**Authors:** Muhammad Furqan, Travis P. Snyders, Mohammed U. Saqlain, Sarah L. Mott, Douglas Laux, Anthony Snow, Carryn M. Anderson, John M. Watkins, Gerald H. Clamon

**Affiliations:** ^1^ Department of Internal Medicine University of Iowa Hospitals and Clinics Iowa City Iowa; ^2^ Biostatistics Core Holden Comprehensive Cancer Center University of Iowa Hospitals and Clinics Iowa City Iowa; ^3^ Department of Pathology University of Iowa Hospitals and Clinics Iowa City Iowa; ^4^ Department of Radiation Oncology University of Iowa Hospitals and Clinics Iowa City Iowa

**Keywords:** chemotherapy, Head and neck cancer, radiation therapy

## Abstract

**Background:**

High‐dose cisplatin (Cis) is a preferred systemic agent for concurrent chemoradiation (CRT) in locally advanced head and neck squamous cell cancer (LAHNSCC) patients. As some patients are unable to tolerate Cis, this study compares the toxicity and efficacy of weekly cisplatin‐paclitaxel (CP) regimen with Cis.

**Methods:**

Patients with LAHNSCC receiving definitive chemoradiation either with Cis (Cisplatin—100 mg/m^2^ q3w x 3) or CP (Cisplatin—20 mg/m^2^; Paclitaxel—30 mg/m^2^qw x7) were included.

**Results:**

Cis and CP groups were comprised of 114 and 111 subjects, respectively**.** Complete response for Cis versus CP groups was 88% versus 88%, respectively. Median follow‐up for the study was 58.5 months. After adjusting for potential treatment selection bias, no significant differences were evident between Cis and CP groups for overall survival (hazard ratios [HR] 0.85, 95% CI 0.59‐1.21, *P* = 0.36), progression free survival (HR 0.88, 95% CI 0.62‐1.24, *P* = 0.46), locoregional control (HR 0.77, 95% CI 0.52‐1.15, *P* = 0.21), and distant control (HR 0.87, 95% CI 0.61‐1.23, *P* = 0.42). Patients in the CP group had less acute and chronic toxicities.

**Conclusions:**

Weekly CP regimen can serve as an alternative systemic therapy with radiation in patients with LAHNSCC who are not fit for Cis.

## 1 INTRODUCTION

Head and neck squamous cell carcinoma (HNSCC) accounts for approximately 3%‐4% of all new cancer cases in the Unites States.^1^ Around 60% of these patients present with locally advanced disease. Meta‐Analysis of Chemotherapy in Head and Neck Cancer (MACH‐NC) has shown improvement in absolute survival by 4.5% with systemic therapy when utilized concurrently with radiation. Therefore these patients are primarily treated with concurrent chemotherapy and radiotherapy (CRT) to preserve organ function. However, an optimal systemic therapy is not well defined. Regimens that were associated with benefit include platinum‐based monotherapy or polychemotherapy regimens containing platinum or fluorouracil (5‐FU) or both.^2^ Due to lack of prospective data, high‐dose cisplatin 100 mg/m^2^ (Cis) monotherapy on days 1, 22, and 43 has been considered the preferred systemic treatment with concurrent radiation. It is an intensive regimen with considerable acute and chronic toxicities including mucositis, nausea, vomiting, myelosuppression, ototoxicity, nephrotoxicity, and neuropathy. A fair number of patients with locally advanced head and neck squamous cell carcinoma (LAHNSCC) are considered “not suitable” for this regimen. Even 30%‐40% of so called “fit” subjects from phase III randomized studies of high‐dose cisplatin could not complete the intended 3 cycles of chemotherapy with radiation.^3^, ^4^, ^5^ Traditionally, alternative platinum‐based regimens or cetuximab have been prescribed to patients who are unfit for high‐dose cisplatin. Wide variation and preference in utilization of these alternative regimens exist among oncologists in the United States as demonstrated by Longitudinal Oncology Registry of Head and Neck Carcinoma (LORHAN) analysis.^6^ The 2 most common substituted regimens are weekly cetuximab and low‐dose cisplatin (30‐40 mg/m^2^). Cetuximab has been found to be inferior to high‐dose cisplatin 7, 8 whereas weekly cisplatin is compared to high‐dose in only small prospective studies or retrospectively.^9^, ^10^, ^11^, ^12^, ^13^, ^14^, ^15^, ^16^, ^17^, ^18^, ^19^, ^20^, ^21^, ^22^

Combination of weekly cisplatin and paclitaxel can be a potential substitute for high‐dose cisplatin and this regimen was developed by RTOG. Weekly cisplatin‐paclitaxel (CP) was effective in a randomized phase II study (RTOG 97‐03) with high rates of compliance.^23^ Despite the fact that combination chemotherapy might be better compared to single agent chemotherapy from neoadjuvant trials of HNSCC,^24^ the combination of CP is significantly underutilized.^6^ In this study, we retrospectively explored the efficacy and toxicity of weekly CP regimen and compared it with Cis when given concurrently with radiation for the treatment of HNSCC.

## 2 MATERIALS AND METHODS

Patients with LAHNSCC who received curative‐intent concurrent chemoradiation from 1 January 1998 to 31 December 2013 were identified from University of Iowa Oncology Registry, a prospectively maintained database of cancer patients. Intended chemotherapy regimen had to be either high‐dose cisplatin 100 mg/m^2^ (Cis) on day 1, 22, and 43 or the combination of cisplatin 20 mg/m^2^—paclitaxel 30 mg/m^2^ (CP) weekly x 7. Patients were excluded if: age <18, nasopharyngeal or salivary gland as the primary site of tumor, 2 different synchronous primaries, presence of distant metastases, and receipt of induction chemotherapy. Study protocol was approved by the Institutional Review Board of University of Iowa. Per institution policy, individual patient consent was not required as this study was retrospective in nature and did not involve any intervention.

Demographic, disease‐, and treatment‐related data were captured through chart review by one of the authors. Comorbidities were scored using the Charlson Comorbidity Index (CCI). Smoking history was defined as “never” smoker if patient smoked <100 cigarette/life time, “former” smoker if quit before development of symptoms from index tumor otherwise regarded as “current” smoker. All patients underwent a thorough staging work‐up which at a minimum included endoscopic examination of aero‐digestive tract, contrast‐enhanced neck CT, and CT of the chest or PET/CT prior to initiation of definitive therapy. Disease sites were categorized as oral, oropharyngeal, hypopharyngeal, larynx, and other primaries based on location of index primary tumor. Toxicities were graded per CTCAE v4.0. Toxicities occurring in the first 90 days from the date of initiation of treatment were considered as acute, and recurrent toxicities were documented only once with highest grade. Late toxicities were recorded at 6, 12, and 24 months from the start of treatment. These included chronic kidney disease, impaired hearing affecting routine activities of daily life, inability to eat solid food, aspiration pneumonia, and neuropathy.

A minimum follow‐up time of 16 months was available for all surviving patients. All patients underwent response assessment after completion of treatment by endoscopic examination and neck imaging either through CT or PET/CT. Complete response (CR) was defined as no evidence of tumor at the site of primary tumor and in the regional nodes. Regional node had to be normal by size criteria or have no increase in FDG uptake on PET/CT at 3 months from treatment completion. Patients undergoing salvage neck dissection for possible residual tumor within 5 months of treatment completion were not considered to have regional recurrence. Loco‐regional failure/recurrence was defined as histologic identification of tumor at site of index tumor at any time after completion of therapy or 5 months after therapy in the regional nodes or death due to index cancer. This definition was adopted from RTOG1016 protocol (NCT NCT01302834). Treatment failure was defined as persistent disease at the site of index tumor after therapy.

To investigate differences in the demographic, clinicopathologic, and outcome variables between intended chemotherapy groups, chi‐squared (Fisher's exact test were appropriate), and Wilcoxon rank sum tests were used. Survival probabilities were estimated and plotted using the Kaplan‐Meier method. For locoregional control, time was calculated from diagnosis to treatment failure, loco‐regional recurrence, or death due to cancer. For distant control, time was calculated from diagnosis to distant recurrence or death due to any cause. For progression free survival, time was calculated from diagnosis to treatment failure, locoregional or distant recurrence, or death due to any cause. For overall survival, time was calculated from diagnosis to death due to any cause. To adjust for potential treatment selection bias, inverse probability score weighted Cox regression models were fit to evaluate differences in outcomes by intended treatment group. Propensity scores were derived from a logistic regression model adjusting for age, smoking status and exposure, ECOG performance status, Charlson Comorbidity Index, baseline laboratory values, primary site, and TN stage. Estimated effects are reported as hazard ratios (HR) along with 95% confidence intervals. All statistical testing was 2‐sided and assessed for significance at the 5% level using SAS v9.4 (SAS Institute, Cary, NC).

## 3 RESULTS

Out of 516 patients treated during the study period, 225 cases met the eligibility criteria of the study. Reasons for exclusion were adjuvant chemoradiation for high risk LAHNSCC (n = 180), receipt of induction chemotherapy (n = 45), use of alternative systemic therapy (n = 50), and others (n = 16). Cis group included 114 patients while CP group composed of 111 patients. Three patients had stage II disease (2 in Cis group and 1 CP group), while remaining patients had stage III or higher disease.

Baseline demographic and clinical data for all patients are summarized in Table 1. Patients prescribed Cis were younger (*P* < 0.01) and more likely to be an active cigarette smoker (*P* < 0.01); however, overall extent of exposure to tobacco was less compared to patients receiving CP (*P* < 0.01). Distribution of patients was balanced with regard to performance status but subjects with higher morbidity (CCI) were more likely to receive CP (*P* = 0.01). Oropharynx was the most common site for the index tumor (75%) in both groups (*P* = 0.35). The mean dose of cisplatin received in Cis and CP groups was 239.0 mg/m^2^ (79.7% of intended) and 120.2 mg/m^2^ (85.8% of intended), respectively. Fifty‐nine (51.8%) patients in the Cis group and 68 (61.3%) in the CP group could not receive all the intended cycles or weeks of chemotherapy (*P* = 0.15). Switch to a different regimen was required in 13.2% versus 2.7% in patients receiving Cis versus CP, respectively. All patients received intensity modulated radiation therapy and completed the intended course of radiation (66‐70Gy) except for 3 patients in Cis and 2 in CP group. Table 2 summarized intended versus delivered therapy.

**Table 1:** Patient characteristics

Variables	Cisplatin (Cis) n = 114 (%)	Cisplatin + Paclitaxel (CP) n = 111 (%)	*P*‐Value
Age (median)	53.0	57.0	**<0.01**
Sex			
Male	84 (73.7)	84 (75.7)	0.73
Female	30 (26.3)	27 (24.3)	
Smoking			
Current	63 (55.3)	38 (34.2)	**<0.01**
Former	30 (26.3)	51 (45.9)	
Never	21 (18.4)	22 (19.8)	
Extent of smoking (only smokers were included)			
≥20 pack‐year	74 (79.6)	87 (97.8)	**<0.01**
<20 pack‐year	19 (20.4)	2 (2.3)	
ECOG performance status			
≤1	108 (94.7)	110 (99.1)	0.12
≥2	6 (5.3)	1 (0.9)	
Charlson comorbidity index (mean)	0.3	0.5	**0.01**
Primary site of SCC			
Oral	2 (1.8)	4 (3.6)	0.35
Oropharynx	85 (74.6)	84 (75.7)	
Hypopharynx	3 (2.6)	5 (4.5)	
Larynx	24 (21.1)	16 (14.4)	
Others	0 (0.0)	2 (1.8)	
Primary site T staging			
T2	64 (56.1)	53 (47.7)	0.33
T3	28 (24.6)	28 (25.2)	
T4	22 (19.3)	30 (27.0)	
TNM staging			
II‐III	21 (18.4)	19 (17.1)	0.80
IVa‐Ivb	93 (81.6)	92 (82.9)	

**Table 2:** Intended vs received therapy

Variables	Cisplatin (Cis) n = 114 (%)	Cisplatin + Paclitaxel (CP) n = 111 (%)	*P*‐value
Completion of prescribed chemotherapy			
No	59 (51.8)	68 (61.3)	0.15
Yes	55 (48.2)	43 (38.7)	
Mean cisplatin‐dose received	239.0 mg/m2	120.2 mg/m2	**<0.01**
Mean % of intended cisplatin dose delivered	79.7%	85.8%	0.12
Switch to a different systemic regimen			
No	99 (86.8)	108 (97.3)	**<0.01**
Yes	15 (13.2)	3 (2.7)	
Carboplatin‐paclitaxel	7	3	
Cisplatin‐Paclitaxel	8	NA	
Receipt of intended radiation dose			
No	3 (2.6)	2 (1.8)	1.00
Yes	111 (97.4)	109 (98.2)	
Break in radiation therapy (≥5 days)			
No	113 (99.1)	107 (96.4)	0.24
Yes	1 (0.9)	3 (2.7)	

All of the 225 patients were included in the efficacy analysis. CR was observed in 100 (87.7%) versus 98 (88.3%) patients in Cis versus CP groups, respectively. Complete response by primary site and nodes in Cis versus CP groups was 104 (91.2%) versus 100 (90%) and 99 (86.8%) versus 96 (86.5%), respectively. Salvage neck node dissection was performed in 1 patient in Cis group and 2 in CP group for residual disease. In Cis group persistent disease was noted in 9 subjects, (primary site only [n = 3], regional nodes only [n = 3], and at both sites [n = 3]). In CP group, 10 subjects had persistent disease (primary site only [n = 2] and at both sites [n = 8]). Response to therapy could not be determined in 5 subjects due to death prior to assessment (n = 4 in Cis group; n = 1 in CP group).

**Figure 1:**
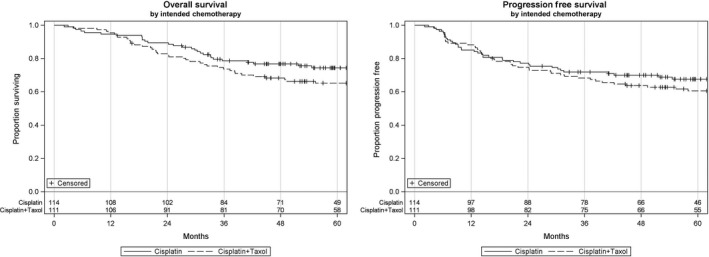
Overall and progression free survival by intended chemotherapy

**Figure 2:**
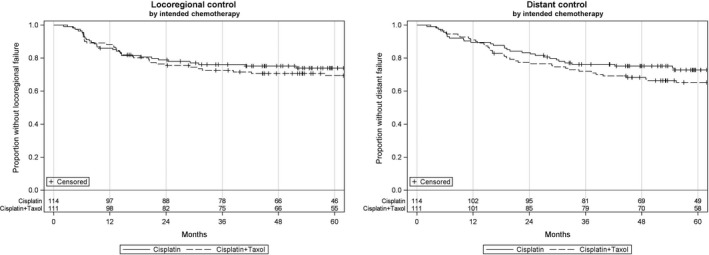
Locoregional and distant control by intended chemotherapy

Median length of follow‐up was 58.5 months (2.1‐186.7 months) for the study. In the Cis group, 12 patients had a locoregional recurrence, 13 patients had a distant recurrence, and 41 patients died. Similarly in the CP group, 13 patients developed locoregional recurrence, 16 patients had a distant recurrence, 48 patients died. Unadjusted Kaplan‐Meier curves along with 2‐ and 5‐year estimates are presented in Figures 1 and 2 and Table 3. After adjusting potential treatment selection bias, no significant differences between the high‐dose cisplatin and CP regimens for overall survival (HR 0.85, 95% CI 0.59‐1.21, *P* = 0.36), progression free survival (HR 0.88, 95% CI 0.62‐1.24, *P* = 0.46), locoregional control (HR 0.77, 95% CI 0.52‐1.15, *P* = 0.21), and distant control (HR 0.87, 95% CI 0.61‐1.23, *P* = 0.42) were evident.

**Table 3:** 2‐ and 5‐year efficacy outcomes according to intended chemotherapy assignment

Outcome	Intended regimen	2 years (95% CI)	5 years (95% CI)
Overall survival	Cisplatin	89% (82%‐94%)	74% (65%‐82%)
	Cisplatin‐Paclitaxel	83% (74%‐89%)	65% (55%‐73%)
Progression free survival	Cisplatin	77% (68%‐84%)	68% (58%‐76%)
	Cisplatin‐Paclitaxel	75% (66%‐82%)	61% (51%‐69%)
Locoregional control	Cisplatin	79% (70%‐85%)	74% (65%‐81%)
	Cisplatin‐Paclitaxel	76% (67%‐83%)	69% (60%‐77%)
Distant control	Cisplatin	83% (75%‐89%)	73% (63%‐80%)
	Cisplatin‐Paclitaxel	65% (55%‐73%)	65% (55%‐73%)

Toxicities of chemotherapy regimens were assessed according to Common Toxicity Criteria of Adverse Events (CTCAE) version 4.0. Due to the retrospective nature of the study, mucositis could not be assessed reliably from patient's electronic records. Among the acute toxicities, patients receiving high‐dose cisplatin had higher incidence of grade ≥ 3 nausea, acute kidney injury (AKI), and presence of ototoxicity, whereas more patients in combination chemotherapy group required feeding tube placement. Distribution of grade 3 or higher vomiting, neuropathy, febrile neutropenia, and any hospitalization was similar between the 2 groups. Assessment of chronic toxicities during 6‐24 months after treatment revealed statistically higher incidence of chronic kidney disease, ototoxicity, and aspiration pneumonia in patients who received high‐dose cisplatin whereas feeding tube dependency was more common in combination chemotherapy group (Table 4).

**Table 4:** Acute and chronic toxicities of treatment

Toxicities	Cisplatin (Cis) n = 114 (%)	Cisplatin‐Paclitaxel (CP) n = 111 (%)		*P*‐Value
Acute	Hospitalization			
	No	69 (60.5)	60 (54.1)	0.33
	Yes	45 (39.5)	51 (45.9)	
	Febrile neutropenia			
	No	104 (91.2)	104 (93.7)	0.48
	Yes	10 (8.8)	7 (6.3)	
	Acute kidney injury			
	Grade ≤ 2	107 (93.9)	111 (100.0)	**0.01**
	Grade ≥ 3	7 (6.1)	0 (0.0)	
	Nausea			
	Grade ≤ 2	85 (74.6)	95 (85.6)	**0.04**
	Grade ≥ 3	29 (25.4)	16 (14.4)	
	Vomiting			
	Grade ≤ 2	93 (81.6)	96 (86.5)	0.32
	Grade ≥ 3	21 (18.4)	15 (13.5)	
	Neuropathy			
	Grade ≤ 2	114 (100.0)	110 (100.0)	
	Ototoxicity (any)			
	No	94 (82.5)	105 (94.5)	**<0.01**
	Yes	20 (17.5)	5 (4.6)	
	Neutropenia			
	Grade ≤ 2	102 (89.5)	106 (95.5)	0.09
	Grade ≥ 3	12 (10.5)	5 (4.5)	
	Thrombocytopenia			
	Grade ≤ 2	114 (100.0)	109 (98.2)	0.24
	Grade ≥ 3	0 (0.0)	2 (1.8)	
	Anemia			
	Grade ≤ 2	110 (96.5)	104 (93.7)	0.33
	Grade ≥ 3	4 (3.5)	7 (6.3)	
	Feeding tube required			
	No	35 (30.7)	6 (5.4)	**<0.01**
	Yes	79 (69.3)	105 (94.6)	
Chronic	Chronic kidney disease			
	No	93 (84.3)	107 (98.2)	**<0.01**
	Yes	16 (14.7)	2 (1.8)	
	Ototoxicity			
	No	86 (80.4)	105 (96.3)	**<0.01**
	Yes	21 (19.6)	4 (3.7)	
	Neuropathy			
	No	102 (95.3)	108 (99.1)	0.12
	Yes	5 (4.7)	1 (0.9)	
	Aspiration pneumonia			
	No	94 (87.9)	107 (99.1)	**<0.01**
	Yes	13 (12.1)	1 (0.9)	
	Feeding tube dependency			
	No	61 (57.0)	34 (31.2)	**<0.01**
	Yes	46 (43.0)	77 (68.8)	

## 4 DISCUSSION

The goal of this study was to compare low‐dose‐cisplatin‐based combination chemotherapy with high‐dose cisplatin in definitive concurrent chemoradiation setting for patients with LAHNSCC. Our findings suggest higher compliance, comparable efficacy, and significantly less acute toxicity with low‐dose CP combination chemotherapy.

Three‐weekly single agent high‐dose cisplatin is considered as the standard of care systemic therapy for definitive concurrent chemoradiation setting. Despite the irrefutable benefits, higher incidence of severe acute toxicities (16%‐47%) and relatively low compliance rates (60%‐70%) raise significant concern regarding tolerability of this regimens in the minds of treating physicians and patients.^3^, ^4^, ^5^, ^25^ In addition, long‐term follow‐up of RTOG91‐11 discovered significantly higher non‐cancer‐related mortality in patients who received high‐dose cisplatin concomitantly with radiation.^26^ Consequently, there has been an effort to reduce the treatment‐related complications without compromising anticancer activity. Weekly low dose cisplatin (30‐40mg/m2) is considered an alternative option to high dose cisplatin as it offers similar dose intensity, reduces chemotherapy‐related acute adverse events, and facilitates dose adjustments according to changes in patient's condition with relatively similar survival benefit as suggested by a meta‐analysis.^25^ Nevertheless, other studies have questioned this finding. Only randomized study comparing low (30 m/m^2^) versus high dose (100 mg/m^2^) cisplatin prospectively found inferior locoregional disease control with low dose cisplatin. While other studies revealed inferior overall survival with low‐dose cisplatin compare to high‐dose cisplatin.^20^, ^27^, ^28^ Despite the controversy of high and low dose cisplatin, 15% of LAHNSCC patients in the United States receive weekly low‐dose cisplatin with radiation according to LORHAN analysis. Cetuximab, an epidermal growth factor receptor monoclonal antibody, is another alternative option to high or low‐dose cisplatin as it is considered to have better side‐effect profile.^29^ However, recent studies have confirmed that it is inferior to high‐dose cisplatin even in HPV positive oropharyngeal LAHNSCC which is a considered as good prognostic disease.^7^, ^8^, ^10^, ^12^, ^14^

Other substitutes to high‐dose cisplatin include combinatorial chemotherapy regimens involving multiple radiosensitizing agents. Combinatorial regimens could disturb varying aspect of tumor biology along with providing systemic effects. Since, survival benefit associated with concurrent chemoradiation is mainly due to improvement in locoregional control; multiagent radiosensitizing regimens may actually do better than single agent cisplatin. This is supported by the reversal in pattern of failure from locoregional recurrences to distant disease as seen in some of the studies that utilized multiagent regimens.^30^, ^31^, ^32^, ^33^, ^34^ MACH‐NC meta‐analysis on the use of chemotherapy also concluded that polychemotherapy either with platin or 5‐FU was not inferior to mono‐platin.^2^, ^35^ However, data comparing combination of relatively newer chemotherapeutic agents with high‐dose cisplatin monotherapy is regrettably lacking. Many centers have attempted to look at weekly carboplatin‐paclitaxel combination in concomitant chemoradiation setting. Not surprisingly, compliance was better and overall outcomes were comparable to historical control.^36^, ^37^, ^38^, ^39^, ^40^, ^41^ But these studies did not directly compare the combination therapy with single agent high‐dose cisplatin (Table 5).

**Table 5:** Outcomes of studies utilizing platinum‐taxol regimens

Primary sites of disease	Retrospective vs prospective	Chemotherapy regimen	Number Of Patients	Overall Survival (OS)	Progression Free Survival (PFS)	Complete response rate (CR)	Ref
All HNSCC	Prospective Single arm	Carboplatin 100 Paclitaxel 40	55	3y‐45% 5y‐35%	3y‐36% 5y‐36%	52%	35
All HNSCC	Prospective Single arm	Carboplatin AUC1 Paclitaxel 60mg/m2	43	NA^†^ 5y‐40%	NA^†^ 5y‐70%	65%	36
All HNSCC	Prospective (UMC0221)	Carboplatin AUC1 Paclitaxel 30 mg/m2	35	3y‐88% NA^†^	NA^†^ NA^†^	NA^†^	37
All HNSCC	Retrospective (two groups –High dose Cis group data nor shown)	Cisplatin 20mg/m2 Paclitaxel 30 mg/m2	111	2y‐83% 5y‐65%	2y‐77% 5y‐61%	88%	Current study
Oropharyngeal (OP) only	Retrospective (one group)	Carboplatin AUC2 Paclitaxel 40	160	3y‐81% 5y‐70%	NA^†^ 5y‐64%	NA^†^	39
All HNSCC	Retrospective (one group)	Carboplatin 100 mg/m2 Paclitaxel 45 mg/m2	60	3y‐48%	3y‐48%	75%	34
HPV + OP	Retrospective (3 groups, weekly Cis, and Cetuximab data not shown)	Carboplatin Paclitaxel	59	3y‐88% 5y‐78%	3y‐84% 5y‐80%	NA^†^	38

^†^ NA: Not available or applicable due to difference in methodology.

Our study aimed to compare a multiagent combination of 2 strong radiosensitizing chemotherapies, cisplatin and paclitaxel, against high‐dose cisplatin to minimize toxicity and improve compliance without jeopardizing tumor control. Outcomes of patients who received CP combination chemotherapy with definitive radiation seems to be numerically inferior at 5‐year time‐point but these differences were not significant. One of the common concerns over low‐dose chemotherapy is their limited efficacy toward eradicating micrometastases affecting distant failure. However, distant control in our study was similar in both the groups. All acute and chronic toxicities that were different between the 2 groups were seen less frequently with combination chemotherapy except for requirement and dependency on feeding tube. Due to retrospective nature of the study, it is unclear why patients in combination chemotherapy group required feeding tube to be placed more often but this may be to prevent malnutrition as patients in this group were old, had higher co‐morbidity and larger primary (T4) tumor compare to high‐dose cisplatin group. Another explanation is patients in combination chemotherapy group had higher degree of swallowing dysfunction. This could suggest higher radiosensitizing potential of the CP regimen. Nonetheless, dependency on feeding tube was different only till 6 months, after that both groups were similar with regards to requiring feeding tube to maintain adequate nutrition.

Our study has several limitations. Due to retrospective nature of the study there is inherent selection bias toward intended chemotherapy assignment for patients. Data pertaining to HPV or p‐16 positivity was lacking for most patients. Since, frequency of oropharyngeal squamous cell carcinoma is similar in both group therefore we believe this should not have affected the results. Toxicity data in our study were captured from patients’ electronic health records and due to differences in documentation among treating physicians, there could be more differences which could not be captured.

## 5 CONCLUSIONS

In patients with LAHNSCC who are not fit for high‐ or low‐dose cisplatin, weekly CP regimen can serve as an alternative systemic therapy with radiation. Our data suggest that it is well tolerated, easy to deliver and have similar efficacy. Our findings need further evaluation through prospective studies.

## CONFLICT OF INTEREST

The authors have no conflict of interest to declare.
